# A Atividade Física e Qualidade de Vida de Crianças com Cardiopatias Congênitas: Uma Questão de Saúde Pública

**DOI:** 10.36660/abc.20230634

**Published:** 2023-10-06

**Authors:** Silvana Vertematti

**Affiliations:** 1 Instituto de Assistência Médica ao Servidor Público Estadual São Paulo SP Brasil Instituto de Assistência Médica ao Servidor Público Estadual (IAMSPE), São Paulo , SP – Brasil; 2 Universidade Federal de São Paulo São Paulo SP Brasil Universidade Federal de São Paulo (UNIFESP), São Paulo , SP – Brasil

**Keywords:** Cardiopatias Congênitas, Doenças Cardiovasculares, Crianças, Qualidade de Vida, Atividade Física, Saúde Pública

A atividade física é tanto uma forma de expressão quanto um meio de promover o desenvolvimento das crianças. A diversão obtida por meio dessa atividade é um dos fatores que mais contribuem para a inserção social da criança, além de garantir a manutenção da sua capacidade de executar movimentos. São inequívocos os ganhos físicos, fisiológicos, psicológicos e sociais que a atividade física traz às crianças e aos adolescentes.

A atividade física se define como qualquer movimento do corpo produzido pelos músculos esqueléticos e que resulta em um gasto de energia. ^1^ O exercício físico é uma atividade física planejada, estruturada dentro de um contexto esportivo e social, com repetições lógicas e organizadas com o objetivo de manter o condicionamento físico. O condicionamento, por sua vez, caracteriza-se por um conjunto de atributos físicos relacionados à melhora da saúde e da performance da criança, e pode ser mensurado por meio de testes físicos específicos realizados por profissionais habilitados. ^1^

A análise do metabolismo das fibras musculares demonstra que as crianças têm um predomínio de fibras do tipo I, com características oxidativas, sobre as fibras do tipo II, mais especializadas no metabolismo anaeróbico. Assim, as crianças possuem uma atividade glicolítica menos eficiente do que a dos adultos. Durante a adolescência, há uma mudança na proporção dos tipos de fibras musculares e uma maturação metabólica que farão com que o indivíduo adquira a composição corporal e as capacidades físicas próprias do adulto. ^2 , 3^

A partir da década de 40, observaram-se melhoras progressivas nas técnicas de correção cirúrgica e de manejo clínico das cardiopatias congênitas. Nesse sentido, os estudos da Blalock e Taussig, que buscaram desenvolver um tratamento para os conhecidos “bebês azuis”, ^4^ contribuíram para que 90% das crianças que nascem atualmente com cardiopatias congênitas tenham boas chances de atingir a idade adulta com excelentes condições clínicas. Entretanto, esses pacientes ainda necessitam de suporte do sistema de saúde para lidar com os eventuais desfechos desfavoráveis, tanto decorrentes da anormalidade congênita propriamente dita, quanto das doenças crônico-degenerativas ocasionados pela falta de atividade física, considerando-se que apenas 19% desses pacientes recebem orientações de atividade física e exercícios físicos formais para combaterem o sedentarismo. ^5 - 7^

A superproteção dos pais e a insegurança tanto de pais quanto de profissionais em prover às crianças uma orientação correta respeito aos exercícios recomendados são algumas das causas dessa situação, além de serem fatores que contribuem para outro problema de saúde pública: a obesidade e suas complicações. Pacientes nessas condições tornam-se vulneráveis à recém descrita tríade da inatividade física pediátrica (TIP, ou “PIT” em inglês), condição que envolve três componentes distintos, porém interrelacionados: (1) o transtorno do déficit de exercício, que acomete os indivíduos que não atingem a recomendação de 60 minutos diários de atividades físicas com intensidade moderada a vigorosa proposta pela Organização Mundial de Saúde para a população pediátrica, (2) a dinapenia, e (3) o analfabetismo físico, caracterizado por uma dificuldade no desenvolvimento das habilidades físicas e da coordenação motora das crianças, criando um ambiente propício para o sedentarismo. ^7 , 8^

As doenças congênitas do coração apresentam um espectro que vai desde defeitos mínimos, que podem passar despercebidos, até condições clínicas complexas, que requerem uma correção cirúrgica precoce. Vários estudos demonstram que as crianças portadoras de malformações complexas sofrem uma diminuição da capacidade funcional, das habilidades motoras e da força muscular decorrente da cianose, do aumento da circulação pulmonar e de intervenções cardíacas. O condicionamento físico e o nível de atividade física são importantes preditores de saúde cardiovascular, e há necessidade de mais estudos que avaliem esses parâmetros na população de crianças e adolescentes. Além dos riscos relacionados ao sedentarismo, esses indivíduos podem apresentar defeitos posturais, como a escoliose e cifose decorrentes da esternotomia realizada durante procedimentos cirúrgicos, além de questões relacionadas à sua qualidade de vida. ^9 , 10^

Em conformidade com as melhoras práticas disponíveis, os médicos e profissionais assistentes devem buscar atingir três objetivos no manejo desses pacientes: (1) transmitir mensagens claras sobre as atividades físicas e suas restrições, (2) seguir as diretrizes de prescrição de exercícios ao paciente e sua família, (3) fornecer um seguimento médico regular para acompanhar a aderência de seus pacientes a suas recomendações, bem como o aumento do seu nível de atividade física e sua tolerância física individual. ^7^

Considerando-se as questões biopsicossociais envolvidas no desenvolvimento do adolescente com uma cardiopatia congênita, faz-se necessário prover um suporte multiprofissional especializado a pacientes nessa fase da vida, para ajudá-los a compreender suas capacidades individuais, seus sentimentos, suas experiências positivas, e também suas frustrações e ansiedades em relação às suas limitações físicas. Além disso, esse suporte deve endereçar as questões motivacionais envolvidas no manejo desses pacientes para promover sua capacidade de adaptação e de avaliação da viabilidade de executarem as atividades que estão dentro de suas capacidades. ^11^

Em suma, variáveis como desempenho nas atividades diárias, capacidade funcional e condicionamento físico, força muscular, postura e qualidade de vida em crianças com cardiopatias congênitas de moderada a severa complexidade devem ser devidamente endereçadas. Além disso, programas de reabilitação assertivos devem ser criados e implementados para contribuir com uma melhora dos padrões de condicionamento físico, fatores determinantes na prevenção de doenças crônico-degenerativas, e que têm grande impacto na saúde pública. ^9^
