# Supplementary material for: A Atividade Física e Qualidade de Vida de Crianças com Cardiopatias Congênitas: Uma Questão de Saúde Pública
Source: Arq Bras Cardiol. 2023 Oct 6;120(9):e20230634. [Article in Portuguese] doi: 10.36660/abc.20230634 (PMC10567081; doi:10.36660/abc.20230634)
Supplement: Translation

# Translation: Physical Activity and Quality of Life in Children with Congenital Heart Diseases: A Public Health Issue

**Authors:** Silvana Vertematti

**Affiliations:** 1 Brasil Instituto de Assistência Médica ao Servidor Público Estadual (IAMSPE), São Paulo, SP – Brasil; 2 Brasil Universidade Federal de São Paulo (UNIFESP), São Paulo, SP – Brasil

**Keywords:** Heart Defects, Congenital, Cardiovascular Diseases, Children, Quality of Life, Physical Activity, Public Health

Physical activity is both a form of expression for children and a means for promoting their development. The fun derived from engaging in this activity is one of the main factors for promoting the social inclusion of children, in addition to ensuring their continued ability to perform movements. The physical, physiological, psychological, and social dividends physical activity brings to children and adolescents are unequivocal.

Physical activity is defined as any bodily movement produced by skeletal muscles and resulting in energy expenditure. ^1^ Physical exercise is a planned physical activity. It is structured within a sports and social context, involves logical and organized repetitions, and is designed to maintain physical fitness. Fitness, in turn, is characterized by a set of physical attributes related to improving the child’s health and performance and can be measured by specific physical tests conducted by trained professionals. ^1^

Analysis of muscle fiber metabolism shows that children have a predominance of type I fibers—which have oxidative characteristics—over type II fibers—more specialized in anaerobic metabolism. As a result, children have less efficient glycolytic activity than adults. Adolescence brings about metabolic maturation and changes in the proportion of muscle fibers that make the individual acquire an adult’s body composition and physical capabilities. ^2 , 3^

As of the 1940s, progressive improvements have occurred in surgical correction techniques and the clinical management of congenital heart diseases. Accordingly, the studies conducted by Blalock and Taussig, which sought to develop a treatment for the so-called “blue babies,” ^4^ contributed to the current state of affairs where 90% of children born with congenital heart defects have a good chance of reaching adulthood with excellent clinical conditions. However, these patients still need the support of the health system to deal with any unfavorable outcomes arising from both the congenital abnormality per se and the chronic-degenerative diseases caused by lack of physical activity, considering that only 19% of them receive guidance on physical activity and formal physical exercises to fight a sedentary lifestyle. ^5 - 7^

Overprotective parents and the insecurity displayed by both parents and professionals in providing children with correct guidance for recommended exercises are some of the causes of this situation, as well as factors that contribute to yet another public health issue: obesity and its complications. Patients in these situations become vulnerable to the recently described pediatric inactivity triad (PIT), a condition that involves three distinct but interrelated components: (1) an exercise deficit disorder, which affects individuals who fail to meet the recommendation of 60 minutes of daily physical activity with moderate to vigorous intensity proposed by the World Health Organization for the pediatric population, (2) dynapenia, and (3) physical illiteracy, characterized by the impaired development of the child’s physical skills and motor coordination, which create an environment conducive to a sedentary lifestyle. ^7 , 8^

Congenital heart diseases range from minimal defects, which may go unnoticed, to complex clinical conditions requiring early surgical correction. Several studies show that children with complex malformations suffer a decrease in functional capacity, motor skills, and muscle strength due to cyanosis, increased pulmonary circulation, and cardiac interventions. The levels of physical conditioning and physical activity are important predictors of cardiovascular health, and further investigation is needed to assess these parameters in children and adolescents. In addition to the risks related to a sedentary lifestyle, these individuals may have postural defects, such as scoliosis and kyphosis, resulting from the sternotomy performed during surgical procedures and issues related to their quality of life. ^9 , 10^

Consistent with the best available practices, physicians and assisting professionals should strive to achieve three goals when managing these patients: (1) convey clear messages about physical activities and their restrictions, (2) comply with guidelines on how to prescribe exercises to patients and their families, (3) provide regular medical follow-up to monitor the patient’s adherence to their recommendations, as well as their increase in physical activity, and individual physical tolerance. ^7^

Bearing in mind the biopsychosocial issues involved in the development of adolescents with congenital heart disease, it becomes apparent that specialized multidisciplinary support should be provided to patients in this phase of life to help them comprehend their capacities, their feelings, their positive experiences, and also their frustrations and anxieties toward their physical limitations. Furthermore, this support should address the motivational issues involved in managing these patients so that they can better adapt and assess the feasibility of performing activities within their capabilities. ^11^

In summary, the variables of performing daily activities, functional capacity and physical fitness, muscle strength, posture, and quality of life in children with moderate to severe congenital heart disease must be properly addressed. In addition, assertive rehabilitation programs must be created and implemented to help improve physical fitness standards, which are determining factors in the prevention of chronic degenerative diseases and which significantly impact public health. ^9^
